# Escitalopram Associated Pancreatitis: A Case Report

**DOI:** 10.1192/j.eurpsy.2026.11063

**Published:** 2026-07-31

**Authors:** S. N. Erdem Cihan, H. Kaya, E. Göka

**Affiliations:** Department of Psychiatry, Ankara Bilkent City Hospital, Ankara, Türkiye

## Abstract

**Introduction:**

Acute pancreatitis (AP) is an acute process of inflammation and cellular injury of the pancreas. Drug-induced pancreatitis (DIP) accounts for approximately 0.1% to 3.4% of acute pancreatitis attacks. DIP should always be considered in recurrent and idiopathic cases.

**Objectives:**

SSRIs have rarely been associated with DIP, whereas the evidence for antipsychotics is largely weak. We aimed to consider psychotropic drugs in cases of acute pancreatitis with unknown etiology.

**Methods:**

Verbal consent has been obtained from the patient for case reporting.

**Results:**

A 79-year-old male patient, who had been using escitalopram 10 mg/day for approximately one year with intermittent adjunctive risperidone 2 mg/day, experienced three episodes of pancreatitis during the same period. He had no history of pancreatitis prior to SSRI initiation. In his most recent episode, elevated lipase (1282 U/L) and amylase (785 U/L) levels were detected, accompanied by typical epigastric pain. Abdominal ultrasound and MRCP showed no evidence of gallstones or sludge. The patient had no history of alcohol use; laboratory results revealed triglyceride level of 56 mg/dL and calcium level of 9.1 mg/dL. In the etiological evaluation, hyperparathyroidism, IgG4-related disease, infection and trauma were excluded. Abdominal CT revealed reticular density increase consistent with acute pancreatitis. In the absence of other medications that could be associated with pancreatitis, DIP was suspected. One week after discontinuation of escitalopram and risperidone, amylase levels decreased to 240 U/L and lipase levels to 383 U/L, with marked clinical improvement. Notably, in previous episodes of pancreatitis, clinical improvement had also been observed even without discontinuation of psychotropic medications.

**Conclusions:**

The diagnosis of drug-induced pancreatitis is most often based on rechallenge with the suspected agent. However, in many cases, the diagnosis can be made solely on the history of drug exposure and exclusion of other etiologies. In this patient, the careful exclusion of alternative causes and the clinical improvement following drug discontinuation support the likelihood of DIP. The Naranjo score was calculated as 5, indicating a “possible” causal relationship. The Badalov classification is used to assess the likelihood of an association between drugs and acute pancreatitis. According to this classification, escitalopram and risperidone are categorized as Class 2 and Class 4, respectively. In our case, the recurrent attacks during long-term SSRI use, the exclusion of other causes and the improvement after drug withdrawal strongly implicate escitalopram as the primary suspect. Risperidone, on the other hand, is considered a concomitant but less likely contributing factor. In unexplained recurrent cases of pancreatitis, SSRIs should always be reconsidered, and clinical/enzymatic responses should be closely monitored following drug discontinuation.

**Disclosure of Interest:**

None Declared

